# Minimally invasive Distal Pancreatectomy eRgonOMic analysis – the DP-ROM trial: An explorative, prospective, observational, cohort study trial

**DOI:** 10.1007/s00464-025-12227-w

**Published:** 2025-10-10

**Authors:** Matteo De Pastena, Salvatore Paiella, Alessandro Esposito, Elisa Venturini, Ermes Vedovi, Massimo Strazzer, Carlo Caselli, Giuseppe Malleo, Gabriella Lionetto, Antonio Pea, Luca Landoni, Elisa Danese, Roberto Salvia

**Affiliations:** 1 University of Verona, Verona, Italy; 2 Pancreatic Surgery Unit, Department of Surgery, Dentistry, Paediatrics and Gynaecology, University of Verona, Verona, Italy; 3 Pancreatic Surgery Unit, University of Verona Hospital Trust, Verona, Italy; 4 Pancreatic Surgery Unit, Department of Engineering for Innovation Medicine (DIMI), University of Verona, Verona, Italy; 5 Recovery and Functional Rehabilitation, Integrated University Hospital of Verona, 37100 Verona, Italy; 6 Zevio, Verona, Italy; 7 Verona, Italy; 8 Section of Clinical Biochemistry, Department of Engineering for Innovation Medicine (DIMI), University of Verona, Verona, Italy; 9 General and Pancreatic Surgery Department, Pancreas Institute, University and Hospital Trust of Verona, Policlinico GB Rossi, Piazzale L.A. Scuro, 10, 37134 Verona, Italy

**Keywords:** Ergonomics, Robotic surgery, Laparoscopic surgery, Pancreatic resection, Musculoskeletal, Injury

## Abstract

**Background:**

Minimally invasive distal pancreatectomy (DP) improves patient outcomes but also introduces significant ergonomic challenges for surgeons. This study quantitatively compares the ergonomic stress and mental workload associated with laparoscopic versus robot-assisted DP.

**Methods:**

An exploratory, prospective, observational cohort trial was conducted with two experienced pancreatic surgeons performing 20 DPs (10 laparoscopic using IMAGE1 S Rubina and 10 robot-assisted using da Vinci® Si platform). Ergonomic stress was assessed using stabilometric analysis, kinematic pelvic movement analysis, ocular examinations, cortisol level monitoring, and the NASA Task Load Index (NASA-TLX).

**Results:**

Robot-assisted DP demonstrated superior ergonomic performance, with reduced physical and mental demands compared to laparoscopic DP. Stabilometric tests indicated significantly greater postural stability during robot-assisted surgery, characterized by lower variance in body oscillations. Kinematic analyses confirmed smoother pelvic movements in robot-assisted procedures, suggesting reduced physical fatigue. Visual strain was markedly higher during laparoscopic surgery, likely due to prolonged screen exposure. The NASA-TLX indicated significantly lower overall workload scores for robot-assisted DP (35.3 ± 19.5) compared to laparoscopic DP (50.7 ± 21.7, *p* = 0.04). Cortisol analyses consistently showed elevated stress levels in the laparoscopic group compared to the robotic group, reinforcing the finding of greater physiological stress in the laparoscopic setting.

**Conclusion:**

Robot-assisted DP significantly reduces ergonomic stress, visual strain, and cognitive workload compared to laparoscopic DP. However, it also introduces specific ergonomic challenges, particularly involving neck and trapezius muscle strain. Future studies should investigate ergonomic modifications to further optimize surgeon well-being and performance during robot-assisted pancreatic procedures.

**Supplementary Information:**

The online version contains supplementary material available at 10.1007/s00464-025-12227-w.

Minimally invasive pancreatic surgery has evolved significantly in recent decades, offering improved patient outcomes compared to traditional open surgery, including reduced postoperative complications, shorter hospital stays, and faster recovery times. When performed by experienced surgeons, both laparoscopic and robot-assisted pancreatic resections demonstrate significant improvements in surgical and postoperative outcomes [1]. However, these benefits are not without cost, though not for patients, but for surgeons themselves. A growing body of evidence underscores the increased ergonomic strain experienced by surgeons performing minimally invasive procedures, with frequent reports of musculoskeletal symptoms affecting the neck, back, shoulders, and hands [2, 3]. These symptoms are often worsened by prolonged operative times and the physical demands intrinsic to the mini-invasive techniques, irrespective of the surgeon’s years of experience [4, 5]. 

While laparoscopic pancreatic surgery poses significant ergonomic challenges, robot-assisted surgery has emerged as a potential solution. The robot-assisted approach enables surgeons to operate from a seated position, thereby reducing the physical strain on the back, shoulders, and neck [6].

However, while the robotic system provides several ergonomic benefits, it is not without its own challenges. Notably, robot-assisted surgery can result in increased activation of specific muscle groups, particularly the trapezius, which may contribute to fatigue and discomfort for the surgeon [6].

In addition to physical strain, surgeons performing minimally invasive procedures encounter visual and cognitive ergonomic challenges. The transition from real-life three-dimensional (3D) vision in open surgery to virtual two-dimensional (2D) or 3D vision during minimally invasive procedures demands significant adaptation [7]. Despite advances in imaging technologies, such as high-definition (HD) and 4 K systems, laparoscopic surgery still requires surgeons to wear specialized goggles to achieve 3D vision, which can lead to dizziness and visual fatigue [8–10]. In contrast, robotic systems offer an integrated 3D visualization system; however, challenges related to visual adaptation and prolonged exposure to screen-based tasks persist and remain a concern for surgeons [11].

Finally, ergonomic stress is not only physical but also psychological, notably due to performance anxiety. Intense mental pressure during complex surgeries can exacerbate physical discomfort [12]. Chronic activation of the hypothalamic–pituitary–adrenal axis raises cortisol levels, contributing to fatigue [13]. Although performance anxiety’s psychological aspects are studied, its direct influence on surgical performance and surgeon well-being remains underinvestigated.

This study aims to evaluate, for the first time in MIPS, the physical, visual, and cognitive ergonomic workloads experienced by surgeons during laparoscopic and robot-assisted distal pancreatectomy (DP). By comparing the workload of the same surgical team across both platforms, we seek to clarify the effectiveness of robot-assisted systems in reducing ergonomic stress, which remains under investigation relative to traditional laparoscopic surgery. We will also examine associated psychological stress and cortisol responses in both approaches.

## Methods

### Participant surgeons

A prospective, exploratory, observational cohort study was conducted. The local Ethics Committee was consulted before the start of the study and stated that no specific ethical approval was required. Two experienced pancreatic surgeons from the General and Pancreatic Surgery Unit of the Integrated University Hospital of Verona, a high-volume center for minimally invasive pancreatic surgery, were enrolled (AE, LL), both with extensive expertise in laparoscopic and robot-assisted DP. Both surgeons were male, right-handed, and aged 47 years old. Each had completed the learning curve for both laparoscopic and robot-assisted DP. The years of surgical practice following residency completion were 15 years. The individual caseload of overall pancreatic procedures, including open procedures, exceeded 1000 cases, while the average number of annual open and minimally invasive DP was 40 and 90, respectively. Both underwent a detailed baseline assessment as described below. The surgeons performed 10 procedures as the first surgeon and 10 as the first assistant at the bedside, changing the role, respectively.

### Stabilometry and postural stability

Surgeons did not receive ergonomic training and were advised to avoid additional physical activity to prevent muscle injuries. Surgeons’ balance and adjustments were assessed using the Lizard Ultimate stabilometric platform (Lizardmed Srl, Monza, Italy), which monitors center of gravity movements. Standardized tests measured the contributions of vestibular, visual, proprioceptive, occlusal, and cervical systems to posture, with loads expressed in kilograms (kg) and percentages (%). Performance was also evaluated with the Wiva Science device (Letsense, Castel Maggiore, Bologna, Italy). This 3-axis accelerometer measures acceleration along three axes, determining the device’s orientation in space for 3D analysis. Equipped with a gyroscope, it tracks all accelerations and decelerations during movement. By calculating the angular range of primary and secondary movements (ROM), it assesses muscle interference and workload-related fatigue. A pre-study physiatric exam screened for recent muscle injuries or physical issues, such as carpal tunnel syndrome or neck/back disorders. All measurements were taken before and after surgery. Additionally, surgeons were asked to repeat the measurements with eyes open and closed to better evaluate postural stability following surgery.

### Ocular stress

An ophthalmologist assessed surgeons for stereoscopic vision and to exclude manifest strabismus using five qualitative and semi-quantitative tests: Lang I and II, Titmus, Bagolini, TNO, and cover/uncover tests. Monocular visual acuity was measured, and both the anterior segment and central fundus were screened for relevant anomalies. Visual defects were also investigated.

The impact of operating theater workload on vision was evaluated with dedicated tests, including direct measurement using the 2WIN infrared binocular autorefractometer (Adaptica srl, Italy). This device is a portable binocular video refractometer and vision analyzer that measures both eyes simultaneously under real-world conditions, detecting refractive errors, eye abnormalities, and vision problems across a range of −15D to + 15D. It also assesses dynamic pupil responses to light and lens centering, identifying myopia, hyperopia, astigmatism, amblyogenic factors, anisometropia, anisocoria, strabismus, and phorias.

Indirect measures of binocular vision were performed using T.N.O. tables, Berens prism sticks, Facchin Card, Wesson Card, and Saladin Card (C.O.I. srl, Termoli, Italy).

### Mental workload

The NASA Task Load Index (NASA TLX) was used to evaluate subjective mental workload [14]. It assessed six domains: mental, physical, temporal, effort, performance, and frustration. Participants rate each domain on a 20-point scale (0 = low, 20 = high), while performing a task. An overall workload score was calculated, with a midpoint threshold for overload set at 49.5 out of 100. For individual subscales, a midpoint threshold of 9.5/20 was used for rounding [15] (Appendix 1).

### Cortisol parameters

Surgeons collected saliva six times daily for baseline values: upon waking, at 8:00 AM, 12:00 PM, 3:00 PM, 7:00 PM, and at 11:00 PM, using Salivette Tubes (Sarstedt, Nümbrecht, Germany). Participants were instructed to avoid alcohol for 48 h, intense exercise for 24 h, and eating, drinking (except water), smoking, or oral hygiene for 1 h before each collection. Intraoperative sampling was performed immediately before the incision, every 2 h during surgery, and at the conclusion. Detailed sample analysis is reported elsewhere [16].

### Operations

Laparoscopic DP was performed using the IMAGE1 S Rubina surgical system (Karl Storz, Tuttlingen, Germany); robot-assisted DP utilized the da Vinci® Si surgical system (Intuitive Surgical, Sunnyvale, CA, USA). Patients with pancreatic body/tail lesions treated with DP were evaluated at the authors’ institution. Laparoscopic DP (LDP) and robotic distal pancreatectomy (RDP) were performed as previously described [17, 18]. In LDP, the patient is placed in the supine position with a 20–25° reverse Trendelenburg tilt and a 15–20° right lateral tilt. The first 12 mm trocar is inserted above the umbilicus for the camera. A 5-mm trocar is then positioned in the epigastrium beneath the left costal margin. A third 5-mm trocar is inserted in the right hypochondrium along the midclavicular line, above the transverse umbilical line. Finally, a 12-mm port is placed in the left hypochondrium, lateral to the umbilicus along the midclavicular line. An additional 5 mm port may be added more laterally in the left hypochondrium to enhance exposure. In RDP, five trocars are used: four 8 mm robotic ports along a transverse line at the umbilicus level (R1 in the right flank, R2 in the right pararectal area, R3—camera—in the periumbilical region, R4 in the left flank), and a 12 mm assistant port positioned below and between R3 and R4. A standard distal pancreatectomy with splenectomy and lymphadenectomy was performed for malignant lesions. For pancreatic body and tail tumors suspected of posterior margin involvement, radical antegrade modular pancreatosplenectomy was performed. When addressing benign tumors, spleen preservation was always attempted. Regarding the technique of transection, the triple-row stapler reinforced with a PGA felt (NEOVEIL Endo GIA Reinforced Reload with Tri-Staple® Technology 60 mm, COVIDIEN, North Haven, CT, USA) was used [19]. The surgical field was drained using a Penrose-type drain placed proximal to the pancreatic remnant. Postoperatively, the drain was managed according to the institutional protocol[20].

### Statistics and sample size

Given the exploratory design, a sample size assessment was not performed. Twenty procedures were deemed sufficient to assess the workload in laparoscopic and robot-assisted DP.

Normally distributed continuous variables were compared using the two-sample t-test (means ± SD). While non-normally distributed variables were analyzed using the Mann–Whitney U test (medians and interquartile ranges, IQR), Categorical variables were assessed using chi-square or Fisher’s exact test (proportions). Statistical significance was set at *p* < 0.05 (two-tailed). Variables with *p* < 0.10 in univariable analysis were included in multivariable regression.

## Results

Twenty patients underwent minimally invasive DP. No spleen-preserving procedures were performed. The final pathology included small ductal adenocarcinoma, intraductal papillary mucinous neoplasm, and neuroendocrine tumor. The median age was 64 [IQR 54–72]. Thirteen patients were female (65%), with a median body mass index of 24 [IQR 22–28]. Seventy percent of the patients had a cystic lesion. The median operative time was 228 min [IQR 180–283], with a median blood loss of 75 cc [IQR 50–200]. No conversion was performed. No vascular resections were performed. No major intraoperative bleeding or significant intraoperative stressors were recorded. Four patients (20%) experienced a postoperative complication; only one patient (5%) developed a postoperative pancreatic fistula.

### Stabilometric and postural analysis

Table 1 summarizes the stabilometric platform results with the operator’s eyes open and closed, showing changes in center of gravity and postural stability. The analysis indicated a forward shift in the center of gravity during both surgical approaches. Body oscillation variance was greater after laparoscopic surgery, especially with eyes closed, suggesting increased postural instability. Movement speed variance, measured by the double plate stabilometer, was higher during robotic surgery compared to laparoscopic, particularly with eyes open. Stabilometric measurements, including speed variance and body oscillation, indicated significantly greater postural instability during laparoscopic procedures. In both “eyes open” and “eyes closed” conditions, the robotic system allowed for more stable postural control.

**Table 1:** Stabilometric and postural analysis during both laparoscopic and robotic surgeries

Surgical approach	Condition	Variance of velocity (VAR V)	Stability (CG shift)
Laparoscopic	Eyes open	High	Significant forward shift
	Eyes closed	Very High	Severe instability
Robotic	Eyes open	Moderate	Moderate forward shift
	Eyes closed	Moderate	Moderate instability

### Dynamic kinematic analysis

Kinematic data from accelerometer and gyroscope recording revealed notable trends in pelvic movement during both surgical surgeries (Table 2). Both the operator and assistant demonstrated a greater and smoother range of pelvic motion during robotic surgery compared to laparoscopic surgery.

**Table 2:** Kinematic data on pelvic movement for both surgical techniques

Surgical approach	Role	Tilt (°)	Obliquity (°)	Rotation (°)
Laparoscopic	Operator	High variability	Increased variability	Erratic movements
Robotic	Operator	Lower variability	Reduced variability	Smooth, consistent movements

### Fatigue and visual strain

Visual strain was significantly higher during laparoscopy during prolonged screen-based tasks (Table 3). In contrast, robotic surgery permitted a more natural seated position, reducing overall physical fatigue but increasing trapezius muscle load due to head positioning at the console.

**Table 3:** Fatigue and visual strain analysis after both laparoscopic and robotic surgeries

Surgical approach	Role	Pre-surgery fatigue	Post-surgery fatigue
Laparoscopic	Operator	Low	High
Robotic	Operator	Low	Moderate

### Mental workload

**Fig. 1:**
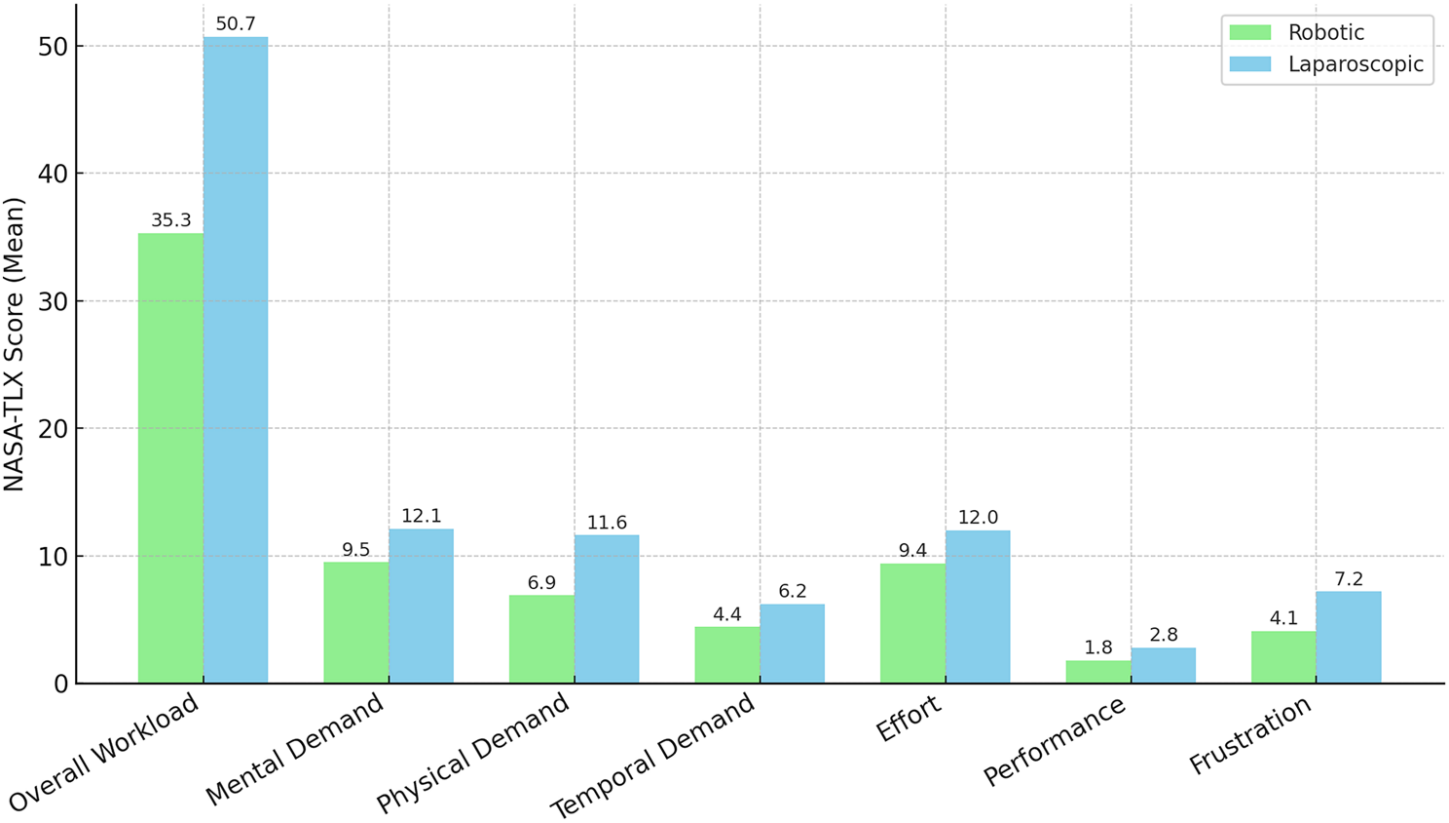
NASA-TLX scores for robotic vs. laparoscopic distal pancreatectomy

Robot-assisted DP was associated with lower overall workload (Table 4). The mean overall workload score for robot-assisted DP was 35.3 (SD ± 19.5), compared to 50.7 (SD ± 21.7) for laparoscopic surgeries DP (p < 0.05). The NASA-TLX workload threshold was exceeded more often in laparoscopic DP (55.9%) compared to robot-assisted DP (25%) (Fig. 1).

**Table 4:** NASA-TLX scores for laparoscopic and robotic distal pancreatectomy

Workload subscale	Robotic surgery (Mean ± SD)	Laparoscopic surgery (Mean ± SD)	P-value
Overall workload	35.3 ± 19.5	50.7 ± 21.7	**0.04**
Mental demand	9.5 ± 5.9	12.1 ± 5.8	**0.03**
Physical demand	6.9 ± 5.2	11.6 ± 5.8	**0.02**
Temporal demand	4.4 ± 4.5	6.2 ± 5.0	0.10
Effort	9.4 ± 6.0	12.0 ± 5.9	**0.04**
Performance	1.8 ± 1.8	2.8 ± 3.5	**0.01**
Frustration	4.1 ± 3.8	7.2 ± 5.6	**0.02**

Bold value indicate statistical significance (p < 0.05)

#### Mental demand

Robot-assisted DP was significantly less mentally demanding than laparoscopic DP. Surgeons rated the mental demand of robot-assisted DP at a mean of 9.5 (SD ± 5.9), compared to 12.1 (SD ± 5.8) for laparoscopic DP (*p* = 0.03).

#### Physical demand

Physical demand was also statistically significantly lower during robot-assisted DP (mean = 6.9, SD ± 5.2) than during laparoscopic DP (mean = 11.6, SD ± 5.8, *p* = 0.02).

#### Temporal demand

Again, robot-assisted DP was rated as less temporally demanding (mean = 4.4, SD ± 4.5) than laparoscopic DP (mean = 6.2, SD ± 5.0), though this difference was not statistically significant (*p* = 0.10).

#### Effort

The effort required during robot-assisted DP was rated significantly lower (mean = 9.4, SD ± 6.0) than that during laparoscopic DP (mean = 12.0, SD ± 5.9, *p* = 0.04).

#### Performance

Performance scores, reflecting surgeons’ self-assessment of task execution, were higher for robot-assisted DP (mean = 1.8, SD ± 1.8, vs. 2.8, SD ± 3.5, *p* = 0.01).

#### Frustration

Frustration levels were significantly lower in robotic surgery (mean = 4.1, SD ± 3.8) than in laparoscopic surgery (mean = 7.2, SD ± 5.6), with a statistically significant difference (*p* = 0.02).

### Cortisol levels and stress response

Table 5 reports cortisol levels during both procedures. Intraoperative cortisol measurements indicated higher physiological stress during laparoscopic DP compared to robot-assisted DP. Hourly sampling demonstrated consistently elevated cortisol levels during laparoscopic DP.

**Table 5:** Cortisol measurements during the laparoscopic and robotic surgeries

	Basal	Robotic first	Robotic second	Laparoscopic first	Laparoscopic second
First surgeon	3.7	3.4	4.6	5.4	2.8
Second surgeon	3.6	2.0	4.8	5.0	2.5

The cortisol levels were express as µg/dL

## Discussion

The results of this study highlight significant differences in ergonomic stress between laparoscopic and robot-assisted DP. Both objective measurements and self-reported fatigue assessments indicate higher physical and visual strain during laparoscopy. The stabilometric analysis reveals a forward shift in the center of gravity in both approaches; however, body oscillation variance is greater during laparoscopy, particularly with eyes closed, suggesting increased postural instability, especially during prolonged procedures. This finding aligns with previous studies that show musculoskeletal strain is associated with laparoscopic surgery due to the need to operate in static, non-ergonomic postures for prolonged periods [21, 22]. In contrast, the robotic system allows surgeons to work from a seated, neutral position, thereby reducing physical strain on the back, shoulders, and neck [23–26]. However, it still requires significant muscle activation, particularly in the trapezius, as the surgeon must maintain a fixed head position at the console [27]. Zihni et al. and Lee et al. already reported that robotic surgery mitigates many of the physical challenges of laparoscopy, although new muscle groups (such as the trapezius) are more actively engaged during robotic procedures [3, 28]. The stability provided by the robotic system is particularly evident in our kinematic analysis, which demonstrates smoother and more consistent pelvic motion during robot-assisted DP. This further supports the hypothesis that robotic surgery facilitates more natural body movements and reduces the fatigue and discomfort commonly experienced during laparoscopy. This ultimately may reduce cognitive load related to maintaining balance and stability, which can otherwise be distracting during laparoscopic surgery.

Visual strain is another area where significant differences have been observed. Laparoscopic surgery typically requires the surgeon to focus on a 2D monitor for extended periods, which can lead to visual discomfort, eye fatigue, and difficulty in maintaining depth perception [29, 30]. These challenges are well-documented and have been linked to increased cognitive load and fatigue, particularly in long procedures. Robotic surgery, however, offers 3D visualization directly through the console, which could alleviate some of the visual strain. While the robotic system provides better visual depth perception, surgeons still encounter a different form of strain, particularly related to the fixed head position, which can lead to neck discomfort after prolonged use [23]. Interestingly, the data from our study indicate that visual discomfort is higher during laparoscopy, aligning with findings from previous studies, which showed that the 3D visualization of robotic surgery is a key factor in reducing visual strain and enhancing surgical performance.

Cognitive workload is higher during laparoscopic surgery, matching the greater physical and visual strain. Surgeons report more fatigue, likely due to the need for intense concentration on a 2D field and suboptimal ergonomics. Robotic surgery, while still demanding, reduces cognitive strain via improved ergonomics, helping surgeons focus on the task rather than posture or vision. Studies confirm robotic surgery consistently lowers cognitive demands, especially for novices [15, 31].

Given the economic and logistical demands of robot-assisted surgery, which will prevent being able to supplant laparoscopy, it is fundamental to continue to seek ways to reduce physical, visual, and cognitive stress in laparoscopy. Optimizing operating table height, using ergonomic instruments, and scheduling regular breaks can help. Postural training may further minimize musculoskeletal stress during long procedures. For robotic surgery, despite clear ergonomic advantages, innovations are still needed to reduce neck strain and improve surgeons’ overall comfort, such as more adjustable and ergonomic consoles. Regular posture and visual comfort assessments, along with fatigue-reducing strategies, can further enhance the long-term benefits of robotic surgery.

This study has some limitations. First, it involved only two surgeons, which does not represent the wider worker population. In fact, the findings may not fully apply to trainees, low-volume surgeons, or female surgeons. Second, the analysis focused on a single type of surgery (DP), which may not include all minimally invasive surgeries. Third, the study lacked long-term follow-up to evaluate the lasting effects of ergonomic stress on surgeons’ health and performance. Chronic musculoskeletal disorders often develop over time, and the short-term analysis used here may not capture the cumulative effects of repeated surgeries. Fourth, environmental factors that could influence the level of visual strain experienced by the surgeon—such as operating room lighting, screen brightness, and the use of magnification systems—were not considered. Finally, the small sample size inherent to its exploratory design limited the statistical power to detect differences between groups. Because of this, we did not apply formal corrections for multiple comparisons, as such adjustments (e.g., Holm–Bonferroni) would significantly increase the risk of type II errors, possibly concealing relevant trends that could inform future research. Our main goal was to generate preliminary, hypothesis-driven data rather than to provide conclusive, confirmatory evidence. However, we recognize that not making adjustments raises the risk of false-positive results, so we interpreted the findings cautiously. Future studies with larger cohorts will be specifically designed to have enough power for proper statistical adjustments to control the family-wise error rate.

## Conclusion

This study shows that robotic surgery significantly reduces physical and cognitive stress for surgeons performing DP compared to laparoscopic surgery. However, robotic surgery also introduces new ergonomic challenges, particularly in the upper back and neck regions. The findings highlight the need to optimize both laparoscopic and robotic setups to enhance surgeon well-being and performance, and call for further research into interventions that minimize musculoskeletal strain and improve comfort during minimally invasive procedures.

## Supplementary Information

Below is the link to the electronic supplementary material.

Supplementary file1 (DOCX 3056 KB)

Supplementary file2 (DOCX 19 KB)

Supplementary file3 (JPG 62 KB)

Supplementary file4 (JPG 57 KB)
