# Supplementary material for: Minimally invasive Distal Pancreatectomy eRgonOMic analysis – the DP-ROM trial: An explorative, prospective, observational, cohort study trial
Source: Surg Endosc. 2025 Oct 10;39(12):8702–9. doi: 10.1007/s00464-025-12227-w (PMC12708784; doi:10.1007/s00464-025-12227-w)

Appendix 1. *The NASA task load index*

The NASA task load index (NASA TLX) is a tool for measuring and conducting a subjective mental workload (MWL) assessment. It allows calculating the MWL of a participant while they are performing a task. It rates performance across six dimensions to determine an overall workload rating. The six dimensions are as follows:

1. Mental demand: how much thinking, deciding, or calculating was required to perform the task.
2. Physical demand: the amount and intensity of physical activity required to complete the task.
3. Temporal demand: the amount of time pressure involved in completing the task.
4. Effort: how hard does the participant have to work to maintain their level of performance?
5. Performance: the level of success in completing the task.
6. Frustration level: how insecure, discouraged, or secure or content the participant felt during the task.

Each subscale is presented to the participants either during or after the experimental trial. They are asked to rate their score on an interval scale ranging from low (0) to high (100).


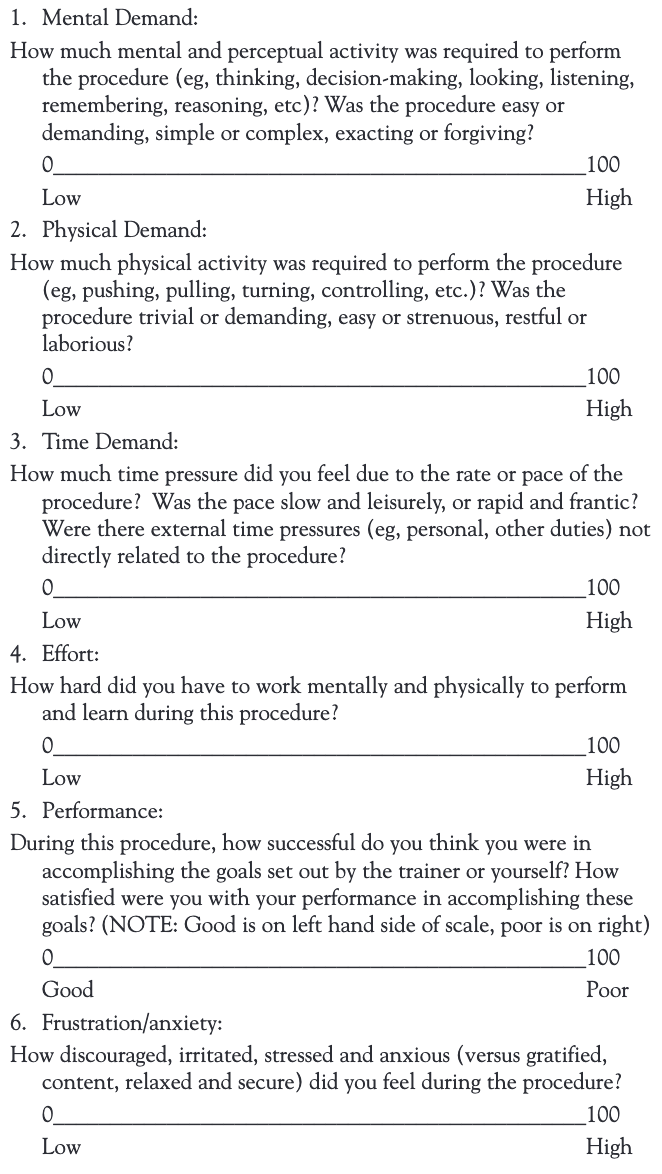

Supplement: Supplementary file 1 — Supplementary file1 (DOCX 3056 KB) [file 464_2025_12227_MOESM1_ESM.docx]
