# Supplementary material for: Minimally invasive Distal Pancreatectomy eRgonOMic analysis – the DP-ROM trial: An explorative, prospective, observational, cohort study trial
Source: Surg Endosc. 2025 Oct 10;39(12):8702–9. doi: 10.1007/s00464-025-12227-w (PMC12708784; doi:10.1007/s00464-025-12227-w)
Supplement: Supplementary file 2 — Supplementary file2 (DOCX 19 KB) [file 464_2025_12227_MOESM2_ESM.docx]

Table S1. Comprehensive stabilometric analysis of variations in the body’s center of gravity and postural stability

|  | **Var V** | | | | | **R Form** | | | | |
| --- | --- | --- | --- | --- | --- | --- | --- | --- | --- | --- |
|  | **Basal** | **Robotic First** | **Robotic Second** | **Lap First** | **Lap Second** | **Basal** | **Robotic First** | **Robotic Second** | **Lap First** | **Lap Second** |
| **First Surgeon**  **OE** | **29.5** | **28.3** | **39.6** | **43.3** | **28.1** | **0.45** | **0.41** | **0.53** | **0.70** | **0.42** |
| **First Surgeon**  **CE** | **43.7** | **47.6** | **51.8** | **50.3** | **46.6** | **0.56** | **0.51** | **0.34** | **0.44** | **0.53** |
| **Second Surgeon**  **OE** | **37.2** | **38.0** | **43.5** | **54.2** | **39.5** | **0.54** | **0.53** | **0.49** | **0.76** | **0.53** |
| **Second Surgeon**  **CE** | **87.6** | **96.8** | **62.2** | **67.9** | **98.8** | **0.37** | **0.35** | **0.43** | **0.43** | **0.35** |
| VAR V: Variance of Velocity; R Form: projection on the sole of the body’s center of gravity; OE: open eyes; CE: closed eyes | | | | | | | | | | |

Table S2. Stabilometric analysis of the left leg variations in the body’s center of gravity and postural stability.

|  | **Var V** | | | | | **R Form** | | | | |
| --- | --- | --- | --- | --- | --- | --- | --- | --- | --- | --- |
|  | **Basal** | **Robotic First** | **Robotic Second** | **Lap First** | **Lap Second** | **Basal** | **Robotic First** | **Robotic Second** | **Lap First** | **Lap Second** |
| **First Surgeon**  **OE** | **47.7** | **41.4** | **25.9** | **23.3** | **45.1** | **0.36** | **0.35** | **0.39** | **0.40** | **0.37** |
| **First Surgeon**  **CE** | **69.23** | **59.8** | **38.7** | **46.2** | **60.7** | **0.35** | **0.33** | **0.40** | **0.44** | **0.37** |
| **Second Surgeon**  **OE** | **59.8** | **60.9** | **41.8** | **47.8** | **61.2** | **0.34** | **0.32** | **0.39** | **0.38** | **0.35** |
| **Second Surgeon**  **CE** | **144.4** | **150.9** | **78.8** | **81.2** | **152.1** | **0.33** | **0.34** | **0.26** | **0.28** | **0.33** |
| VAR V: Variance of Velocity; R Form: projection on the sole of the body’s center of gravity; OE: open eyes; CE: closed eyes | | | | | | | | | | |

Table S3. Stabilometric analysis of the right leg variations in the body’s center of gravity and postural stability.

|  | **Var V** | | | | | **R Form** | | | | |
| --- | --- | --- | --- | --- | --- | --- | --- | --- | --- | --- |
|  | **Basal** | **Robotic First** | **Robotic Second** | **Lap First** | **Lap Second** | **Basal** | **Robotic First** | **Robotic Second** | **Lap First** | **Lap Second** |
| **First Surgeon**  **OE** | **17.0** | **29.9** | **38.2** | **39.8** | **21.2** | **0.42** | **0.37** | **0.32** | **0.33** | **0.36** |
| **First Surgeon**  **CE** | **36.2** | **34.8** | **56.2** | **49.8** | **39.4** | **0.36** | **0.32** | **0.28** | **0.29** | **0.35** |
| **Second Surgeon**  **OE** | **36.5** | **40.1** | **54.9** | **49.8** | **41.3** | **0.38** | **0.32** | **0.30** | **0,28** | **0.33** |
| **Second Surgeon**  **CE** | **99.5** | **97.0** | **118.7** | **123.4** | **98.2** | **0.34** | **0.32** | **0.29** | **0.23** | **0.35** |
| VAR V: Variance of Velocity; R Form: projection on the sole of the body’s center of gravity; OE: open eyes; CE: closed eyes | | | | | | | | | | |

Figure S1. Gait analysis of Surgeon 1 after robotic procedure. The blue line is the left leg. The red line is the right leg. A) Preoperative analysis; B) Second surgeon analysis after robotic procedure; C) First surgeon analysis after robotic procedure

Figure S2. Gait analysis of Surgeon 2 after robotic procedure. The blue line is the left leg. The red line is the right leg. A) Preoperative analysis; B) Second surgeon analysis after robotic procedure; C) First surgeon analysis after robotic procedure
