# Supplementary figures and images for: Minimally invasive Distal Pancreatectomy eRgonOMic analysis – the DP-ROM trial: An explorative, prospective, observational, cohort study trial
Source: Surg Endosc. 2025 Oct 10;39(12):8702–9. doi: 10.1007/s00464-025-12227-w (PMC12708784; doi:10.1007/s00464-025-12227-w)

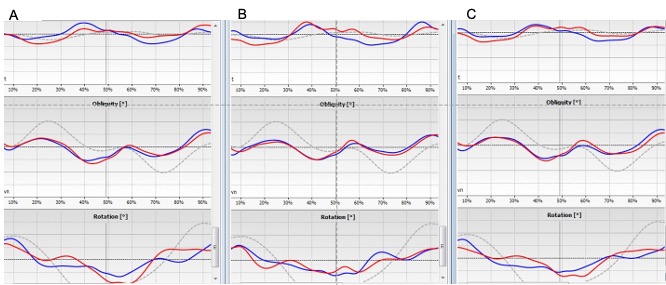

Supplement: Supplementary file 3 — Supplementary file3 (JPG 62 KB) [file 464_2025_12227_MOESM3_ESM.jpg]

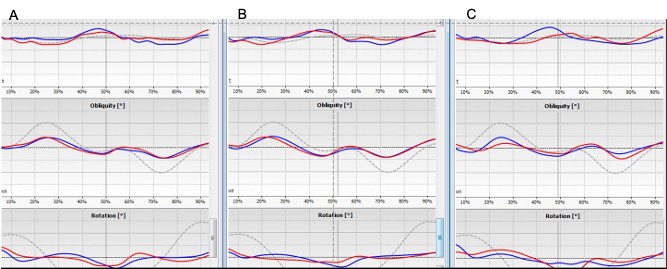

Supplement: Supplementary file 4 — Supplementary file4 (JPG 57 KB) [file 464_2025_12227_MOESM4_ESM.jpg]
